# Progress towards 2020 global HIV impact and treatment targets

**DOI:** 10.1002/jia2.25779

**Published:** 2021-09-21

**Authors:** Mary I. Mahy, Keith M. Sabin, Ali Feizzadeh, Ian Wanyeki

**Affiliations:** ^1^ Strategic Information Department UNAIDS Geneva Switzerland

**Keywords:** AIDS‐related deaths, HIV, models, new HIV infections, UNAIDS 90‐90‐90 targets

## Abstract

**Introduction:**

Over the past 20 years, the response to the HIV epidemic has achieved remarkable results. These results have often been motivated by targets adopted by countries through United Nations (UN) Political Declarations on HIV. The 2016 political declaration included two impact targets, to achieve a 75% decline in new HIV infections and AIDS‐related deaths between 2010 and 2020, and to reach the 90‐90‐90 testing and treatment targets by 2020. Our objective is to summarize progress towards these targets using robust and comparable HIV estimates released by UNAIDS in July 2021. In addition, we comment on the importance of targets and the modelled estimates required to quantify those targets.

**Discussion:**

The UNAIDS estimates indicate that at the global and regional levels, the 2020 targets were missed: new infections declined by 31% and AIDS‐related deaths declined by 47% between 2010 and 2020, compared to a target of 75% decline for both indicators. Similarly, no region achieved the 90‐90‐90 testing and treatment targets. Some countries, in diverse settings, achieved these targets showing that the targets were not overly ambitious if the right funding, policies and evidence‐informed interventions at the right scale were in place. The 2021 UN Political Declaration on HIV, adopted on 8 June 2021, has set out a new set of ambitious but achievable targets for 2025. The 2025 targets and the required actions to reach those targets are described in the Global AIDS Strategy 2021–2026, which provides a framework to reprioritize HIV responses by reducing inequalities and building on the achievements of multiple Sustainable Development Goals. The Strategy encourages countries to monitor progress against targets for different geographic areas and populations to maximize equitable services and ensure accountability and also to understand why targets are being missed.

**Conclusions:**

The UNAIDS epidemiological estimates provide information that promote accountability and estimate progress towards global targets at the national level. Additional strategic information and analyses are required to identify the populations that are furthest from the targets and the programmes and policies that are keeping countries from meeting their targets.

## 1 INTRODUCTION

Since the first AIDS diagnoses in the early 1980s [1], the HIV response has produced remarkable achievements from community involvement in the response, to price negotiation with pharmaceutical companies, to scaling up treatment to over 27.5 million [uncertainty bounds (UB) 26.5–27.7 million] of the 37.7 million (UB 30.2–45.1 million) people living with HIV [2]. The lessons from these achievements allow countries to adopt evidence‐based strategies and plot a clear trajectory towards ending the AIDS epidemic in the coming decade [3, 4, 5]. HIV‐related innovations have also transformed the current COVID‐19 pandemic response, with direct links to lessons from the HIV response [6].

One of the key factors in the success of the HIV response is the global commitment to ambitious targets, renewed every 5 years, that focus national responses on effective interventions and drive accountability. The 2016 Political Declaration on HIV included targets to reduce new HIV infections and AIDS‐related deaths by 90% between 2010 and 2030, with a milestone target of 75% reduction between 2010 and 2020 [7]. Additionally, the 90‐90‐90 testing and treatment targets were established: 90% of people living with HIV know their status, 90% of people who know their HIV‐positive status are receiving treatment and 90% of those people on treatment have suppressed viral loads. Additional targets were set to highlight the importance of prevention interventions and social enablers to end the AIDS epidemic [7].

The UNAIDS estimates released in July 2021 provide comparable estimates of countries’ progress towards the targets set in 2016. The country, age and sex‐specific estimates show that there was improvement in reducing new HIV infections and AIDS‐related deaths and scale up in the testing and treatment targets in some settings; however, the targets were missed globally and at regional levels. There are still geographic areas and populations where gains have not been realized and new HIV infections and AIDS‐related deaths are increasing – despite globally recognized interventions and policies to prevent new HIV infections and avoid AIDS‐related deaths. This commentary summarizes the changes in new HIV infections and AIDS‐related deaths and the scale‐up towards the testing and treatment targets and recognizes the importance of the UNAIDS modelled estimates in quantifying those targets.

## 2 DISCUSSION

New HIV infections, AIDS‐related deaths and the testing and treatment indicators are difficult to measure directly and the quality of country systems to report on these data varies, making comparisons challenging. Every year, UNAIDS supports countries to produce modelled epidemiological estimates of key HIV indicators, including new HIV infections, AIDS‐related deaths and testing and treatment indicators. These estimates ensure that countries (1) have a clear understanding of the impact of HIV on their population, (2) can describe trends and the trajectory of their epidemic, (3) can measure the impact of the HIV response, (4) can measure progress towards targets and (5) can inform planning by identifying the populations and geographic locations with the largest gaps towards reaching the targets. The UNAIDS estimates are comparable across countries and are produced by country teams ensuring ownership of the results and that the most appropriate data are used to inform models [8]. (The country and regional estimates are publicly available at aidsinfo.unaids.org, in the UNAIDS Global AIDS Update Report and on the UNAIDS website. More information about the process of developing estimates and specifically about changes to the 2021 estimates is available in the methods section of the annual Global AIDS Update Report. [2].)

The UNAIDS epidemiological estimates show that globally new HIV infections declined by 31% and AIDS‐related deaths by 47% between 2010 and 2020. And, as of December 2020, an estimated 84% (UB: 67 to >98%) of people living with HIV knew their status, 87% (UB: 67–98%) of people who knew their HIV‐positive status were receiving antiretroviral treatment and 90% (UB: 70–98%) of those on treatment were virally suppressed. While it is encouraging to see that the third testing and treatment target was met, this is only among those who know their HIV status and are on treatment. Viral suppression among all people living with HIV was 66% (UB: 53–79%) in 2020. The wide uncertainty bounds in the global testing and treatment estimates reflect the missing programmatic data from some countries and the modelled uncertainty of the estimates of people living with HIV.

Reviewing the targets for sub‐populations shows important gaps. Globally, women (15 years and older) had a sharper decline in AIDS‐related deaths than men (15 years and older), and women and men had similar declines in new HIV infections. Children (0–14 years) had steeper declines in new HIV infections and AIDS‐related deaths primarily due to the successful rollout of treatment to pregnant women living with HIV (Table 1). Globally, children are far behind adults for the testing and treatment targets reaching only 59‐91‐75 compared with 85‐87‐91 among adults [UB for children: 59% (UB: 42–74), 91% (UB: 63–98), 75% (UB: 52–96) and for adults: 85% (UB:68 to >98), 87% (UB: 67 to >98), 91% (UB: 70 to >98)]. Globally, children are much less likely to be diagnosed than adults, and even when on treatment, their viral load suppression is considerably lower than adults [9]. In most countries, men also show less progress in achieving the testing and treatment targets (82‐83‐91) than women (88‐90‐91) [9] [UB for men: 82 (UB: 65 to >98), 83 (UB: 64 to >98), 91 (UB: 70 to >98) and women: 88 (UB: 71 to >98), 90 (UB: 70 to > 98), 91 (UB: 70 to >98)].

**Table 1:** Progress towards 2020 target of a 75% reduction in new HIV infections and AIDS‐related deaths by the end of 2020 and the 2020 estimates, by UNAIDS region

Region	New HIV infections 2020	Percent change from 2010	AIDS‐related deaths 2020	Percent change from 2010	Testing and treatment targets
Global	1.5 million (1.0–2.0 million)	–31%	680,000 (480,000–1,000,000)	–47%	84‐87‐90
Children 0–14 years	150,000 (100,000–240,000)	–53%	99,000 (68,000–160,000)	–59%	59‐91‐75
Women 15+ years	660,000 (450,000–920,000)	–28%	240,000 (170,000–360,000)	–51%	88‐90‐91
Men 15+ years	640,000 (460,000–890,000)	–27%	340,000 (230,000–490,000)	–37%	82‐83‐91
Asia Pacific	240,000 (170,000–310,000)	–12%	130,000 (87,000–200,000)	–37%	76‐84‐96
Caribbean	13,000 (8700–18,000)	–28%	6000 (4300–8500)	–51%	82‐82‐89
Eastern and southern Africa	670,000 (470,000–930,000)	–43%	310,000 (220,000–470,000)	–50%	89‐87‐91
Eastern Europe and central Asia	140,000 (120,000–160,000)	+43%	35,000 (28,000–43,000)	+32%	70‐76‐94
Latin America	100,000 (66,000–150,000)	+2%	31,000 (20,000–46,000)	–19%	80‐81‐92
Middle East and North Africa	16,000 (12,000–28,000)	+7%	7900 (6000–13,000)	–17%	61‐71‐87
Western and central Africa	200,000 (130,000–330,000)	–37%	150,000 (100,000–210,000)	–43%	77‐95‐81
Western and central Europe and North America	67,000 (53,000–81,000)	–11%	13,000 (9200–17,000)	–30%	90‐93‐89

Note: For regional definitions and country‐specific estimates, please visit aidsinfo.unaids.org.

No region achieved the targeted 75% declines, and two regions, eastern Europe and central Asia and Middle East and North Africa, actually experienced increases in new infections (Latin America had a minor increase in new infections). Eastern and southern Africa was estimated to have the largest decline in new HIV infections (43%) and the Caribbean achieved the largest decline in AIDS‐related deaths (51%), while eastern Europe and central Asia had the largest increase in both new HIV infections (43%) and AIDS‐related deaths (32%) (Table 1). Partial progress was made towards achieving some of the testing and treatment targets with six of the eight regions achieving at least one or more of the targets.

At the country level, two of 128 countries with available estimates had reduced new HIV infections by 75% between 2010 and 2020 and only three of 131 countries reduced AIDS‐related deaths by 75% over the same time period [9] (limited to countries with more than 50 new HIV infections or AIDS‐related deaths in 2010). By the end of 2020, at least eight countries had achieved the 90‐90‐90 testing and treatment targets. Achievement of the testing and treatment targets by countries as diverse as Botswana and Switzerland (e.g. in terms of income level, type of epidemic and healthcare system) demonstrates that countries with drastically different settings can achieve these targets [10].

It is important that countries also examine variations within subnational geographic areas. Results from the UNAIDS‐supported Naomi model [11] show the considerable variations by geographic area within countries. Although the Naomi model does not provide trends over time or the full testing and treatment targets, the available estimates emphasize the diversity within countries: in 20 of 39 countries with available district‐level estimates in 2020, there was a 10‐fold difference in HIV incidence among women 15–24 years between the highest and lowest incidence districts [2].

The poor performance of some populations, countries and regions to achieve these targets suggests that more focused and cost‐effective responses are urgently required. Disaggregation of estimates are useful to identify which populations and geographic locations are missing the targets.

Missing from these 2020 results are estimates of new HIV infections, AIDS‐related deaths and the testing and treatment targets for key populations at increased risk of HIV (men who have sex with men, sex workers, people who inject drugs, transgender persons and prisoners). Key populations are the majority of people living with HIV in most countries outside of the very high burden countries in eastern and southern Africa [2]. Given their importance in HIV epidemics, it is critical to reach key populations with prevention and treatment services if countries are to reach their national targets. However, stigma, discrimination and even criminalization of key populations limit people's willingness to report behaviours that put them at increased risk to HIV. This in turn limits programme managers ability to collect and analyse data on services for key populations. As a result, there is little systematic data to identify key populations reached with HIV services the relevant prevention and treatment cascades, and the associated gaps. The data that do exist from multiple studies, across many settings, show lower diagnoses rates among key populations [12, 13, 14, 15]. Eliminating stigma and discrimination faced by key populations and revising punitive laws are important steps towards reaching the global targets [16].

In June 2021, world leaders from 165 countries adopted the “Political Declaration on HIV and AIDS: Ending Inequalities and Getting on Track to End AIDS by 2030” [17]. The Global AIDS Strategy 2021–2026 lays out the framework and targets to reach the targets of the Political Declaration and to put the world on track to end AIDS by 2030 [18]. The Strategy addresses differential achievements through the adoption of an inequalities lens to refocus the HIV response [18] and recognizes the evidence that key populations as well as some age groups consistently faced disadvantages in accessing HIV and other health services [19]. By committing to target‐specific age and gender groups, key populations and geographic areas, countries create an accountability framework that ensures all members of their society are equitably served by HIV prevention and treatment services to ultimately end AIDS by 2030. The targets within the Strategy also reflect this inequality lens and are linked closely to other Sustainable Development Goals, such as reducing inequality and empowering women and girls, which if reached, will leverage countries’ ability to reach the HIV targets.

In the global health arena, and particularly within the HIV response, country commitments to targets have motivated countries to strive to improve their response and reach the ambitious targets and remain accountable to those commitments. A clear example of promoting accountability is the UNAIDS 90‐90‐90 testing and treatment targets [20]. Countries adopted these targets into their national strategic plans [3, 21, 22] and donor organizations promoted these targets within their funding agreements [23, 24]. These testing and treatment targets were translatable to country‐level actions, including national campaigns to scale‐up testing and treatment programmes [25, 26, 27, 28]. The remarkable support and adoption of the 90‐90‐90 targets shows that when targets are embraced, routinely measured and published, they can motivate countries to act. Reflecting this success, the 2021 Political Declaration and the new Global AIDS Strategy include the more ambitious 95‐95‐95 targets. The availability of these estimates and the ability to quantify progress towards targets also empower communities to advocate for appropriately focused responses.

The UNAIDS epidemiological estimates are an important tool for countries to report on the targets. However, producing estimates for granular populations and locations also requires more data and more assumptions and must be interpreted with caution, recognizing the limitations of the data used to create the estimates. Estimates and targets at very granular levels must always be considered with their respective uncertainty bounds, which reflect the quantity and quality of data input to the model for the specific country and the uncertainty in the parameters used in the model. These summary cross‐sectional targets should complement in‐depth analyses to understand the causes of HIV incidence and mortality declines. Finally, the considerable lack of data for key populations requires additional resources to be dedicated to compiling these data through surveys and programmatic data in a confidential and respectful manner and in collaboration with affected communities.

Modelled estimates have helped produce critical estimates of indicators by age, sex and geographic location in countries with high‐quality data available. Some of the remaining challenges in achieving (and measuring) the targets for key populations include unequal access to critical services and stigma and discrimination [29, 30, 31]. Understanding the complexities underlying inequities and how to capture them in models will be a necessary future step to improve efficiencies as models continue to inform the HIV response. Other inequality dimensions, such as wealth, education status and ethnicity, are currently not captured in most epidemiological models because indicators are often not available by these characteristics.

The estimates process requires national estimates teams to review the quality of their surveillance, size estimation and programme data on an annual basis before entering it into the models. Through this review process, inconsistencies and gaps in the required data have been identified resulting in increased efforts to develop data systems that support the collection of these data. Further investment in national and subnational surveillance surveys, routine prevalence data, health information systems and improving population size estimates will be required to further improve the granularity of estimates. Improved data going into the models will reduce the uncertainty in the estimates, which are so critical for setting policies and agendas and evaluating HIV programmes.

## 3 CONCLUSIONS

The epidemiological estimates released by UNAIDS in 2021 provide a summary of progress against commitments to the 2016 UN Political Declaration. Although progress was made, especially around the testing and treatment targets, there are still important gaps in all regions. With approximately 9 years remaining to reduce annual new HIV infections and prevent AIDS‐related deaths by 90% and achieve the Sustainable Development Goal HIV target, countries need to close the remaining gaps in prevention and treatment efforts and remove the societal barriers to these services. The 2021 Political Declaration sets the targets and describes the services, systems, societal enablers and policies needed to close the gaps for different populations. Policy decisions and programme coverage focusing on eliminating inequalities and preventing new HIV infections and AIDS‐related deaths are vital to achieving those targets. Producing modelled estimates by age, sex, key population and geographic location will play a critical role in supporting countries to identify, monitor and address gaps efficiently.

## COMPETING INTERESTS

The authors have no competing interests.

## AUTHORS’ CONTRIBUTIONS

MIM drafted the manuscript. KMS, AF and IW provided critical feedback and helped shape the messaging of the commentary. All authors are employees of UNAIDS who produce the estimates described in this commentary.
